# Repertoire Sequencing of B Cells Elucidates the Role of UNG and Mismatch Repair Proteins in Somatic Hypermutation in Humans

**DOI:** 10.3389/fimmu.2019.01913

**Published:** 2019-08-27

**Authors:** Hanna IJspeert, Pauline A. van Schouwenburg, Ingrid Pico-Knijnenburg, Jan Loeffen, Laurence Brugieres, Gertjan J. Driessen, Claudia Blattmann, Manon Suerink, Danuta Januszkiewicz-Lewandowska, Amedeo A. Azizi, Marcus G. Seidel, Heinz Jacobs, Mirjam van der Burg

**Affiliations:** ^1^Department of Immunology, Erasmus Medical Center, University Medical Center Rotterdam, Rotterdam, Netherlands; ^2^Laboratory for Immunology, Department of Pediatrics, Leiden University Medical Center, Leiden, Netherlands; ^3^Department of Pediatric Oncology and Hematology, Erasmus Medical Centre, Sophia Children's Hospital, Rotterdam, Netherlands; ^4^Department of Pediatric and Adolescent Oncology, Gustave Roussy Cancer Campus, Villejuif, France; ^5^Department of Paediatrics, Juliana Children's Hospital, Haga Teaching Hospital, The Hague, Netherlands; ^6^Department of Pediatric Hematology and Oncology, Palliative Care, Olgahospital Klinikum Stuttgart, Stuttgart, Germany; ^7^Department of Clinical Genetics, Leiden University Medical Center, Leiden, Netherlands; ^8^Department of Pediatric Oncology, Hematology and Transplantology, Poznan University of Medical Sciences, Poznań, Poland; ^9^Department of Pediatrics and Adolescent Medicine, Medical University Vienna, Vienna, Austria; ^10^Research Unit Pediatric Hematology and Immunology, Division of Pediatric Hematology-Oncology, Department of Pediatrics and Adolescent Medicine, Medical University Graz, Graz, Austria; ^11^Division of Tumor Biology and Immunology, The Netherlands Cancer Institute, Amsterdam, Netherlands

**Keywords:** B cells, somatic hypermutation, DNA repair, mismatch repair (MMR), base excision repair (BER), immunoglobulin, B-cell receptor, constitutional mismatch repair deficiency (CMMRD)

## Abstract

The generation of high-affinity antibodies depends on somatic hypermutation (SHM). SHM is initiated by the activation-induced cytidine deaminase (AID), which generates uracil (U) lesions in the B-cell receptor (BCR) encoding genes. Error-prone processing of U lesions creates a typical spectrum of point mutations during SHM. The aim of this study was to determine the molecular mechanism of SHM in humans; currently available knowledge is limited by the number of mutations analyzed per patient. We collected a unique cohort of 10 well-defined patients with bi-allelic mutations in genes involved in base excision repair (BER) (*UNG*) or mismatch repair (MMR) (*MSH2, MSH6*, or *PMS2*) and are the first to present next-generation sequencing (NGS) data of the BCR, allowing us to study SHM extensively in humans. Analysis using ARGalaxy revealed selective skewing of SHM mutation patterns specific for each genetic defect, which are in line with the five-pathway model of SHM that was recently proposed based on mice data. However, trans-species comparison revealed differences in the role of PMS2 and MSH2 in strand targeting between mice and man. In conclusion, our results indicate a role for UNG, MSH2, MSH6, and PMS2 in the generation of SHM in humans comparable to their function in mice. However, we observed differences in strand targeting between humans and mice, emphasizing the importance of studying molecular mechanisms in a human setting. The here developed method combining NGS and ARGalaxy analysis of BCR mutation data forms the basis for efficient SHM analyses of other immune deficiencies.

## Key Points

- Patients with MMR or UNG deficiency have reduced frequency and altered patterns of SHM in line with a five-pathway model to generate SHM.
- Trans-species comparison identifies differences in strand targeting of SHM between man and mice.

## Introduction

The formation of high-affinity antibodies is based on the efficient introduction of somatic point mutations in the variable region of the B-cell receptor (BCR) encoding genes. Initiation of these somatic hypermutations (SHM) requires the activity of activation-induced cytidine deaminase (AID) that deaminates cytosines (C) to uracils (U), thereby generating highly mutagenic U lesions (1, 2). Establishing mutations at or around the initial U lesion requires error-prone processing involving the activity of the conventional DNA repair proteins uracil-DNA-glycosylase (UNG) and proteins from the mismatch repair (MMR) pathways (3).

Mutations in *UNG* are very rare and cause the immunodeficiency hyper-IgM type 5 (OMIM #608106). Due to a defect in class switch recombination (CSR), these patients mainly produce IgM, leading to recurrent opportunistic infections. In contrast, bi-allelic mutations in MMR result in constitutional mismatch repair deficiency (CMMRD) (OMIM #276300). This is a rare childhood cancer predisposition syndrome without overt clinical signs of an immunodeficiency (4).

To determine the role of UNG and MMR in SHM, knock-out and knock-in mice with defined mutations in these genes have been used. Thereby, three main pathways have been identified to resolve the U lesions introduced by AID (5, 6). First, if B cells replicate before resolving the U lesion, the U is recognized as a template T by the replicative polymerases resulting in C>T and G>A transitions. Second, the base excision repair (BER) enzyme Ung removes the U generating an apyrimidinic site (AP) (7). Upon subsequent cell division, translesion synthesis (TLS) polymerases including Rev1 are recruited, which can bypass AP sites (8). Since AP sites are non-instructive, any nucleotide can be inserted across from them, resulting in transitions and transversions at GC base pairs. Third, the U lesion can be recognized as a U:G mismatch by the MMR binding complex Msh2/Msh6, leading to the activation of exonuclease 1 (Exo1), which removes a stretch of nucleotides leaving a single-strand DNA gap (9, 10). Subsequently, site-specific monoubiquitination of proliferating cell nuclear antigen at lysine 164 (PCNA-Ub) facilitates a polymerase switch from a replicative polymerase (POLD or POLE) to POLH, which preferentially inserts mismatched nucleotides opposite T nucleotides specifically at WA/TW motifs (8, 11–17).

More recently, the existence of a fourth Ung+Msh2 hybrid pathway was proposed, which requires both the single-strand gap generation by Msh2/Msh6 and the AP generation by Ung (8, 10). In this pathway the U:G mismatch is recognized by the Msh2/Msh6 complex, and a single-strand gap is created by Exo1. If, however, on the opposite strand an AP site is created by Ung, TLS can insert a base opposite of the AP site resulting in transversions at template CG base pairs (18). Additionally, a fifth long patch BER pathway has been proposed, which is independent of Msh2, but dependent on Ung, PCNA-Ub, and POLH and accounts for 10–20% of mutations at AT base pairs (5, 14).

Although, a lot is known about the mechanism of SHM in mice, it is still not completely clear what the roles of Pms2 and Mlh1 are in SHM. They were long thought to be dispensable for SHM (19–23); however, a recent publication by Girelli Zubani et al. showed that Ung/Pms2 double knockout mice have a 50% reduction in the number of mutations at AT base pairs (24). They suggest that the Pms2/Mlh1 complex provides the nick required for AT mutagenesis and that in the absence of the Pms2/Mlh1 complex, Ung can compensate for its function.

Virtually all studies that focused on elucidating the molecular mechanism of SHM were performed in mice. Very few studies have been able to study the role of UNG and MMR proteins in SHM in humans as deficiencies in *UNG* or MMR are very rare. So far, three studies have been able to analyze the SHM spectrum in the VH3-23 region of IGHM transcripts of purified CD19+CD27+ B cells using Sanger sequencing in human *MSH6* deficiency (four patients, mean: 103 mutations), *UNG* deficiency (two patients, mean: 119 mutations), or *PMS2* deficiency (two patients; 65 mutations on average) (25–27). In this study, we have been able to collect a unique cohort of 10 patients carrying bi-allelic mutations in *UNG, MSH2, MSH6*, or *PMS2*. In these patients, we performed next-generation sequencing (NGS) of the BCR heavy chain (IGHG and IGHA transcripts), allowing more in-depth analysis (mean 2,842 mutations per patient). The obtained data set allowed us to test if and to what extent the SHM models described above are applicable to humans. Furthermore, we compared our results to published results from different knockout mouse models and patients. These trans-species comparisons provided the first insights into differential activities of defined mutator pathways in establishing the mutation spectra.

## Methods

### Healthy Controls and Patients

Peripheral blood samples were collected from one *MSH2* patient, three *MSH6* patients, five *PMS2* patients, and one *UNG* patient (Table 1). From the UNG-deficient and the MSH2-deficient patient, peripheral blood was obtained with informed consent according to the guidelines of the Medical Ethics Committees of the Erasmus MC. From patient MSH6-01 and PMS2-05, frozen PBMCs were obtained from the Dutch Childhood Oncology group (DCOG or SKION in Dutch) from project number OC2016-009. The remainder of the patients were recruited *via* the network of human geneticists and (pediatric) oncologists working throughout Europe and the Middle East who were informed about the study at conferences and *via* personal communication from 2014 to 2017. Most of participating physicians were partners of the consortium “Care for CMMRD (C4CMMRD)” (4). This study was performed in compliance with current guidelines for good clinical practice and the Declaration of Helsinki with an IRB approval (29-178 ex 16/17) from the Medical University Graz (IRB00002556). Data of the included age-matched HCs have been previously published by IJspeert et al. (32) (see Supplemental Table 1).

**Table 1:** Characteristics and B cell levels of the CMMRD- and UNG-deficient patients.

**Patient code**	**Study number (4)**	**Age range in years**	**Gene**	**Mutations**	**CD19/μl^a^**	**CD19+/CD27+/IgD– (csBm) %B^a^**
MSH2-01		4–5	*MSH2*	c.1147C > T (p.R383X); large deletion including MSH2	860 (700–1,300)	0.5 (3.9–16.2)
MSH6-01		11–18	*MSH6*	c.651dupT (p.K218X); c.3957dupA (p.A1320SfsX5)		
MSH6-02 (4)	13	6–10	*MSH6*	c.1135_1139delAGAGA (p.R379X); c.2277_2293del (p.E760PfsX6)	195 (300–500)	2.26 (3.85–16.5)
MSH6-03 (4)	14	6–10	*MSH6*	c.2238dupT (p.L747SfsX9); c.2980T > A (p.Y994N)	582 (300–500)	5.54 (3.85–16.5)
PMS2-01 (4, 28)	15	11–18	*PMS2*	c.2007-2A > G;^§^ c.2007-2A > G	168.5 (300–500)	5.37 (4–22.8)
PMS2-02 (4, 29)	11	6–10	*PMS2*	c.2444C > T (p.S815L); c.2444C > T (p.S815L)	548 (300–500)	4.52 (3.85–16.5)
PMS2-03 (4)	5	6–10	*PMS2*	c.634C > T (p.Q212X); c.1239del (p.D414TfsX34)	193 (300–500)	9.88 (3.85–16.5)
PMS2-04 (4)	16	6–10	*PMS2*	c.1145-31_1145-13del;^§§^ c.1145-31_1145-13del	213 (300–500)	10.47 (4–22.8)
PMS2-05		11–18	*PMS2*	c.943C > T (p.R315X); c.943C > T (p.R315X)		
UNG02		19–61	*UNG*	c.392delC (p.P131HfsX13); c.392delC (p.P131HfsX13)	200 (300–500)	0.7 (6.5–29.1)

§ *In a different patient, it was shown that the mutation c.2007-2A > G leads to the following two aberrant transcripts: r.2007_2023del (p.S669RfsX9) and r.2007_2174del (p.S669_A725delinsR)*.

§§ *cDNA sequencing showed two aberrant transcripts: r.1144_1145insGATAGTCCACGTTTGCTTAG (p.N383X) and r.1145_2006del (p.G382VfsX19)*.

a *Reference values are indicated in brackets and were taken from Huck et al. (30) for the age groups 4–5 years, 6–10 years, and 11–18 years; and from Warnatz and Schlesier (31) for the age group 19–61 years*.

### Repertoire Sequencing Using NGS

PBMCs were isolated from peripheral blood samples using Ficoll. For analysis of the antigen-selected BCR repertoire, mRNA was isolated from total PBMCs using the Gen-Elute Mammalian total RNA miniprep kit from Sigma-Aldrich (St. Louis, MO). The cDNA libraries were derived from 2 μg of RNA using the Superscript II reverse transcriptase kit from Invitrogen (Paisley, UK). IGH rearrangements were amplified in a multiplex PCR using the forward VH1-6 FR1 (BIOMED-2) primers and either the CgCH1 or the IGHA reverse primer (33–35). Afterwards, PCR products were purified and sequenced using Roche 454 sequencing as previously described (36). In short, PCR products were purified by gel extraction (Qiagen, Valencia, CA) and Agencourt AMPure XP beads (Beckman Coulter, Fullerton, CA). Subsequently, PCR products were quantified using the Quant-it Picogreen dsDNA assay (Invitrogen, Carlsbad, CA). The purified PCR products were sequenced on the 454 GS junior instrument using the Lib-A or Lib-A V2 kits. For one patient, 100 ng of DNA was used to amplify VH-JH rearrangements using VH1-6 FR1 forward primers (BIOMED-2) and JH consensus reverse primers (33). The PCR product was purified and sequenced as described above. This patient (PMS2-01) was excluded from the analyses of subclass distribution as no information on the constant domain can be obtained using this approach. In addition, this patient is not included in the calculation of the percentage SHM as, in contrast to the other patients and the HCs, the sequences obtained from this patient are not selected for IGHA and IGHG and therefore also contain all sequences from naïve and/or non-switched cells thereby greatly influencing SHM levels.

### Immune Repertoire Data Analysis

**Figure 1:**
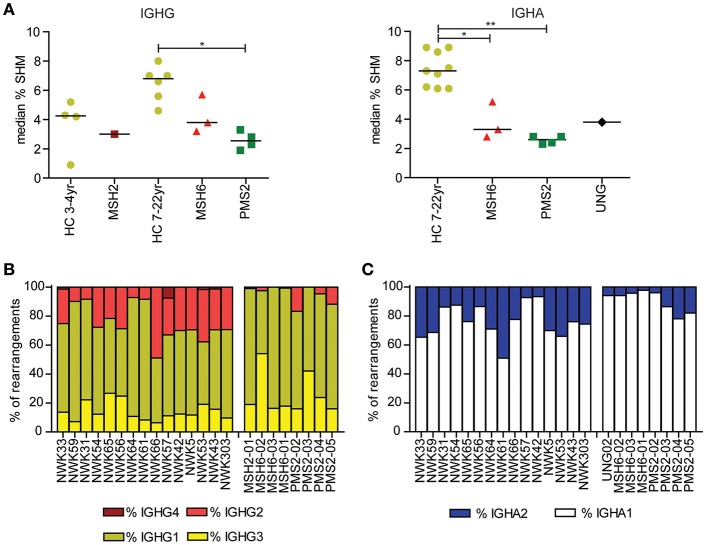
Mutations in MMR and BER proteins lead to reduced frequency of SHM. **(A)** Median SHM frequency in IGHG and IGHA rearrangements is reduced in UNG-, MSH2-, MSH6-, and PMS2-deficient patients compared to HCs. Frequency of IGHG **(B)** and IGHA subclasses **(C)**. Patients with UNG-, MSH2-, MSH6-, and PMS2-deficiency have more IGG1, IGG3, and IGA1 rearrangements compared to HCs. Statistical significance was performed using a Mann–Whitney test and indicated using ^*^P < 0.05 and ^**^P < 0.01.

**Figure 2:**
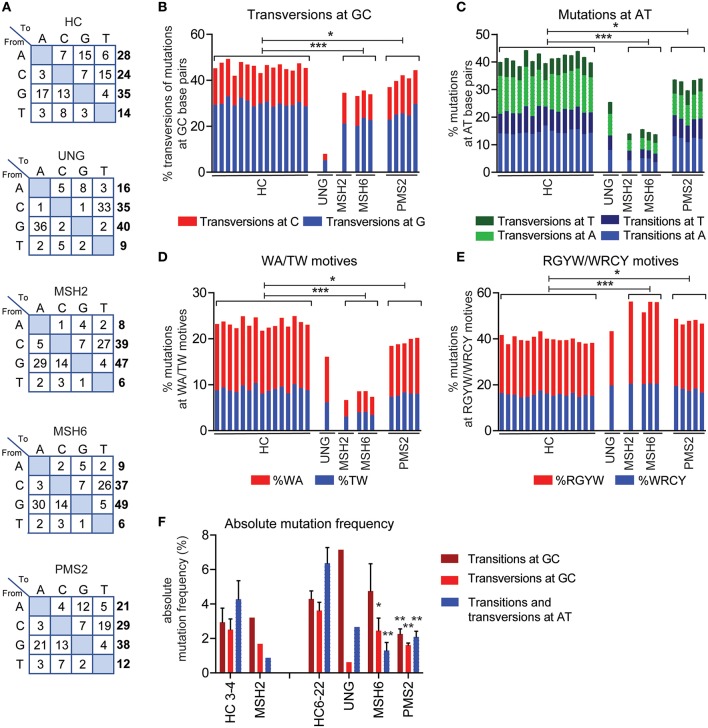
UNG and MMR deficiency result in changes in SHM patterns. **(A)** Transition tables of HCs, UNG-, MSH2-, MSH6-, and PMS2-deficient patients. The percentage of mutations at GC base pairs that are transversions **(B)** and the percentage of mutations at AT base pairs **(C)** in HCs and UNG-, MSH2-, MSH6-, and PMS2-deficient patients. The percentage of SHM present in WA/TW **(D)** and RGYW/WRCY motifs **(E)** in HCs and patients with genetic defects in MMR or BER. **(F)** Absolute frequency of mutations at AT base pairs and transitions and transversions at GC base pairs as compared to age-matched controls (*n* = 5 for age 3–4 and *n* = 10 for age 6–22). Statistical significance was performed using a Mann–Whitney test and indicated using ^*^P < 0.05, ^**^P < 0.01, and ^***^P < 0.001.

**Figure 3:**
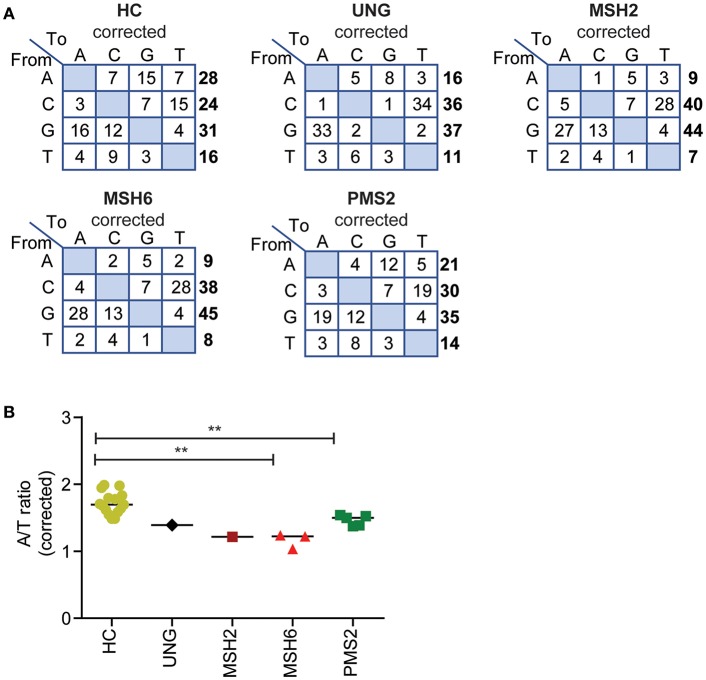
Defects in UNG, MSH2, MSH6, and PMS2 influence the corrected A/T ratio. **(A)** Transition tables of the percentage of mutations corrected for the A,C,T, and G content of the sequenced transcripts. **(B)** The corrected A/T ratio in HCs and patients with genetic defects in *UNG, MSH2, MSH6*, and *PMS2*. Statistical significance was performed using a Mann–Whitney test and indicated using ^**^P < 0.01.

**Figure 4:**
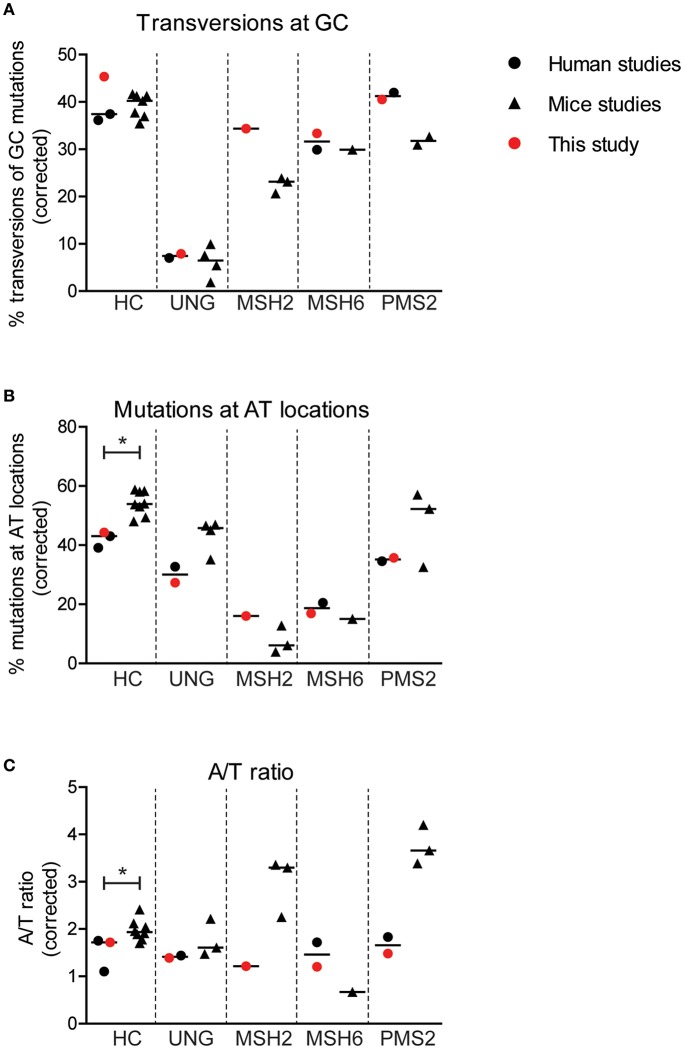
Minor differences are found in SHM patterns between mice and human. Differences in the percentage of GC mutations that are transversions **(A)**, the percentage mutations at AT base pairs **(B)**, and the A/T ratio **(C)** between mice and human with and without genetic defects in genes involved in BER or MMR. Statistical significance was performed using a Mann–Whitney test and indicated using ^*^P < 0.05.

Sequences were demultiplexed based on their multiplex identifier sequence and trimmed from both sides (40 nt for sequences obtained from RNA and 30 nt for the sequences obtained from DNA) to remove the primer sequence using ARGalaxy (37). Fasta files were uploaded in IMGT/High-V-Quest (version 1.5.6) (38), and subsequently the IMGT output files were analyzed using ARGalaxy (37). Here, incomplete sequences or sequences containing an ambiguous “n” base were excluded. To remove sequencing errors, only sequences of which the exact sequence was present at least twice were included. Afterwards, any duplicate sequences were removed. Due to high clonal relation of the sequences in a subgroup of patients, which could lead to major skewing in SHM patterns, only one sequence per clone (based on V-gene and maximal 3-nt difference within the CDR3 using Change-O) (32, 39) was included in further analysis. From the filtered sequences, information on mutation patterns and targeting motifs were obtained. For the analysis of SHM levels and subclass distribution, one sequence per clone based on Class, V-gene, and maximal 3-nt difference within the CDR3 using Change-O (39) was included and results were split into IGHA and IGHG as it is known that these parameters differ between these classes (32). All HC samples were re-analyzed using the same analysis settings. The number of reads after filtering and the number and patterns of mutations (transition tables) can be found in Supplemental Table 1. Samples containing a low number of reads for either IGHA or IGHG after filtering (<45) were excluded from the analysis of SHM levels and subclass distribution (Supplemental Table 1). All calculations used for Figures 1–**4** can be found in the Supplemental Methods.

### Literature Search

On the 19th of March 2018, we search PubMed for publications on Ung, Msh2, Msh6, and Pms2 deficiency in mice and humans using the following search criteria: (((((msh2[Title/Abstract]) OR msh6[Title/Abstract]) OR pms2[Title/Abstract]) OR mlh1[Title/Abstract]) OR UNG[Title/Abstract]) AND (shm[Title/Abstract] OR somatic hypermutation[Title/Abstract]). We excluded reviews, publications without transition tables or information about the nucleotide composition of the sequenced region, and publications in which the SHM was measured in cell lines or in the switch regions. Eight publications matched these criteria (8, 18, 19, 21, 24, 40–42). In addition, we received a transition table on three of the four MSH6 patients from the Gardes et al. publication, which were also included in the analysis (26). The publications on the UNG-, MSH6, and PMS2-deficient patients did not contain information on the nucleotide composition of the sequenced region (25, 27). In both publications, only the IGHV3-23 gene was analyzed for SHM; therefore, we calculated the nucleotide composition of the VH3-23 (FR1–FR3) region ourselves (21% A, 22% T, 25% C, 32% G) and included these in the analysis. For calculations of the number of transversions at GC base pairs, the number of mutations at AT base pairs, and the A/T ratio, we corrected the number of mutations for the nucleotide composition because most publications sequenced a different genomic region to analyze SHM. An overview of the publications and data used to calculate the mutations can be found in Supplemental Table 2.

### Data Visualization and Statistics

Plots were made using GraphPad Prism V7.0, and statistics on these plots were done using the same software.

## Results

### Patients With BER and MMR Deficiency Have Reduced Frequency of SHM and Altered Subclass Usage

To study the molecular mechanism of SHM in human, we included a unique cohort of 10 patients with UNG, MSH2, MSH6, or PMS2 deficiency, and 15 previously published age-matched HCs (Table 1) (32). Since the amount of blood that was available for most of the patients was very limited, we decided to analyze the IGHA and IGHG rearrangements from total PBMCs. These rearrangements are derived from antigen-experienced B cells that have undergone CSR and SHM. For the majority of patients (*n* = 8), we were able to analyze both IGHA and IGHG rearrangements. For the MSH2- and UNG-deficient patients, <45 IGHG (UNG) or IGHA (MSH2) sequences were obtained, and therefore these samples were excluded from the analysis of the SHM frequency or subclass distribution of this class. From one PMS2 patient (PMS2-01), only DNA from PBMCs was available; therefore, we sequenced the IGH rearrangements on the DNA level precluding subclass assignment and the analysis of SHM levels.

Recently, we have shown that patients with CMMRD have no overt clinical immunodeficiency (4). In this study, we were able to include data from three additional CMMRD patients. In line with the study of Tesch et al. the frequency of SHM was reduced in both IGHG and IGHA rearrangements, except for one of the MSH6-deficient patients (MSH6-01) in which SHM was within the normal range for IGHG rearrangements (Figure 1A). Despite the fact that the frequency of switch memory B cells was within the normal range in most of the patients (Table 1), the distribution of the IGHG and IGHA subclasses was different in the CMMRD- and UNG-deficient patients. They had an increased frequency of IGHG3, IGHG1, and IGHA1 compared to the HCs (Figures 1B,C). Thus, although patients with CMMRD have no clinical signs of immunodeficiency, molecular analysis of the BCR rearrangements shows clear aberrancies.

### Analysis of SHM in UNG- and MMR-Deficient Patients Shows Evidence for Five SHM Pathways in Human

According to current models based on knockout mice, Ung is crucial in generating transversions at GC base pairs and the Msh2/Msh6 complex is necessary to generate mutations at AT base pairs. In line with this model, the UNG-deficient patient had a ~4.5-fold decrease in the frequency of transversions at GC base pairs (Figures 2A,B, Supplemental Table 1). In addition, the MSH2- and MSH6-deficient patients had an almost threefold decrease in the frequency of mutations at AT base pairs and WA/TW motifs, suggesting that ~65% of the AT mutations are MSH2/MSH6 dependent (Figures 2A,C,D). Additionally, the MSH2- and MSH6-deficient patients had a significant increase in the frequency of mutations in RGYW/WRCY motifs, confirming that the MSH2/MSH6-dependent pathway creates mutations outside RGYW/WRCY motifs (Figure 2E).

In line with the five-pathway model, we also observed ~35% less mutations at AT base pairs and WA/TW motifs in the UNG-deficient patient (Figures 2A,C,D), suggesting that, in humans, UNG is necessary to create the remaining ~35% of the mutations at AT base pairs. The MSH2- and MSH6-deficient patients also had a small but significant decrease in the frequency of mutations at GC base pairs that are transversions (Figures 1A,B), confirming that in humans, the MSH2/MSH6 complex is also necessary to generate ~25% of the transversions at GC base pairs, possibly *via* the proposed UNG/MSH2 hybrid pathway. Details on the number of bases sequences and the number of mutations can be found in Supplemental Table 1.

To get more insight into the absolute changes, we also analyzed the absolute mutation frequency (mutations/number of sequenced AT or CG bases). This revealed that UNG deficiency leads not only to a strong reduction in transversions at GC base pairs but also to a reduction of the mutations at AT base pairs (Figure 2F), confirming the role of UNG in generation GC transversions and a minority of AT mutations. The absolute number of GC transitions was increased in the UNG-deficient patient, suggesting that in the absence of UNG, more U lesions persist and serve as a template for DNA synthesis.

In the MSH2- and MSH6-deficient patients, the absolute number of mutations at AT base pairs was significantly reduced (Figure 2F), which also explains at least part of the reduced frequency of SHM since almost half of all mutations locate at AT base pairs. Furthermore, a significant reduction in the absolute number of GC transversions was observed in these patients, indicating a critical contribution of the mismatch recognition complex MSH2/MSH6 in generating GC transversions. Altogether, these data show that in humans the U:G mismatches can be resolved *via* direct DNA synthesis across U, UNG-dependent short patch BER pathway, the UNG/POLH-dependent long-patch BER pathway, the UNG-independent MSH2/MSH6-dependent pathway, and the UNG/MSH2 hybrid pathway.

### The Role of PMS2 in SHM

Pms2 has long been thought to be dispensable for SHM in mice. Also, in two patients with PMS2 deficiency, no differences in the mutation spectrum were reported (27). In this study, we were able to study 254–1,388 unique IGH rearrangements per patient in a total of five PMHS2-deficient patients. In the single previous study, only two PMS2-deficient patients and seven unique B cell clones could be studied (27). The increased sequencing depth revealed clear differences in the mutation spectrum. The PMS2-deficient patients had a ~1.2-fold decrease in the frequency of AT mutations and mutations in TW/WA motifs (Figures 2A,C,D). Furthermore, they had a ~10% decrease in transversions at GC base pairs, and a ~1.2-fold increased frequency of mutations in RGYW/WRCY motifs (Figures 2B,E). This was in line with the trends found in the MSH2- and MSH6-deficient patients, although the differences were much milder. The absolute mutation frequency pattern is different in the PMS2-deficient patients compared to the MSH2- and MSH6-deficient patients (Figure 2F). The mutation pattern from PMS2-deficient patients showed a reduction in the number of AT mutations (68%) and transversions at GC base pairs (56%), but they also revealed a decrease in the number of GC transitions (53%). Altogether, these data indicate that PMS2 contributes to the error-prone resolution of U:G mismatches in humans, although its exact role remains to be determined.

### PMS2, MSH2, and MSH6 Deficiency Result in an Altered A-Over-T Mutation Ratio

The ratio between mutated A's and T's gives information about the targeting of the non-transcribed vs. the transcribed strand as PPOLH has in intrinsic T over A mutation bias (a four times higher mutation frequency when copying T's then A's. Because the number of sequenced A's and T's in our data was not exactly the same (24.7–25.9% A's, 20.8–21.5% T's), we first corrected the number of mutations at A's and T's for the number of sequenced A's and T's to calculate the A/T ratio (Figure 3A, Supplemental Methods). In the HCs, the average A/T ratio was 1.7 (range 1.5–2) (Figure 3B), indicating that the non-transcribed strand is mutated ~3 times more than the transcribed strand, leading to a 3:1 strand bias (24, 43).

The A/T ratio in the UNG-deficient patient was not different from the HCs (Figure 3B) (24). However, in the MMR-deficient patients, the A/T ratio was significantly decreased (Figure 3B). The average A/T ratio in the MSH2- and MSH6-deficient patients was 1.2 (range 1–1.2), which corresponds to a 3:2 ratio of error-prone repair by POLH on the non-transcribed vs. the transcribed strand. This suggests that in the absence of the MSH2/MSH6 complex, the A/T bias is almost lost and error-prone repair at AT pairs is more skewed toward the transcribed strand. The PMS2-deficient patients had a decreased A/T ratio of 1.5 (range 1.4–1.5), indicating a 2:1 bias of mutations toward the non-transcribed strand.

### Mutation Spectrum of Human and Mice Indicates Differences in Strand Targeting

To check how conserved the molecular mechanism of SHM is, we decided to perform a trans-species comparison between our human data and previously published data on *Ung, Msh2, Msh6*, and *Pms2* knockout mice, and the data on the previously published UNG- and PMS2-deficient patients. Eleven publications in which transition tables and information on the nucleotide composition of the sequenced region were present (see Methods for details) were included (8, 18, 19, 21, 24–27, 40–42). The frequency of transversions at GC base pairs was comparable between human and mice in both the control and the UNG-deficient patients (Figure 3A). However, the number of transversions seemed slightly lower in the Msh2, Msh6, and Pms2 knockout mice, compared to the patients (Figure 4A).

Interestingly, the average frequency of mutations at AT base pairs was significantly lower in the human controls compared to the wild-type mice (42 and 54%, respectively) (Figure 4B). This suggest that, in mice, more U:G mismatches are resolved by the MSH2/MSH6/EXO1 and the UNG/POLH pathways. The frequency of mutations at AT base pairs was lower in the UNG-deficient patients compared to the Ung knockout mice, but likely relates to an overall decreased mutation frequency at AT base pairs in humans. The reduction of mutations at AT base pairs in the MSH2-, MSH6-, and PMS2-deficient patients was comparable to the knockout mice, suggesting that the role of these proteins is similar in human and mice. The A/T ratio in the human controls was slightly lower compared to wild-type mice (median 1.7 vs. 1.9), while the UNG-deficient patients and mice had a similar A/T ratio (Figure 4C). In contrast, major differences are found in the A/T ratio between the MSH2- and PMS2-deficient patients and Msh2 and Pms2 knock-out mice (24, 43). In summary, these trans-species comparison identified differences in strand targeting of SHM between man and mice, although more research needs to done to understand the exact mechanism.

## Discussion

The induction of SHM in the BCR genes is essential to create high-affinity antibodies. Until now, the molecular mechanism of SHM was mainly studied using mouse models. The studies in humans were very limited because of the rarity of the patients, and the number of unique rearrangements that could be studied was very low. This study is the first of its kind in which a unique cohort of patients with rare diseases affecting DNA repair are combined and analyzed using high-throughput sequencing of the BCR rearrangements.

UNG deficiency results in an immunodeficiency, but patients with CMMRD do not have an overt immunodeficiency (4). Still, most of the CMMRD patients have reduced absolute numbers of B cells (Table 1). Interestingly, the frequency of SHM was reduced in virtually all patients. The absolute mutation frequencies (Figure 2F) showed that this reduction in SHM was mostly caused by a reduction of the mutations at AT base pairs, which account for almost half of the SHM, and a reduction of transversion mutations at GC base pairs. The transition mutations at GC base pairs remained the same in the MSH2 and MSH6 patients, suggesting that the replication pathway does not compensate for the loss of the MMR proteins.

The number of switch memory B cells was normal in most of the patients (Table 1); however, the frequency of IGHG3, IGHG1, and IGHA1 was increased in the CMMRD- and UNG-deficient patients (Figure 1). These rearrangements contain constant genes that are closest to the VDJ exon and arise more frequently from direct switching compared to the more downstream-located constant genes (44). Increased usage of these proximal constant genes can be related to a defect in CSR as switching to the more downstream-located constant genes often requires multiple recombination events. This can have different causes, like a disturbed B–T cell interaction, a proliferation defect, as well as defects in CSR itself. Mouse studies have shown a role for UNG and MMR in CSR (5), but from our data alone, it is impossible to determine the underlying cause of the altered subclass distribution observed (42).

**Figure 5:**
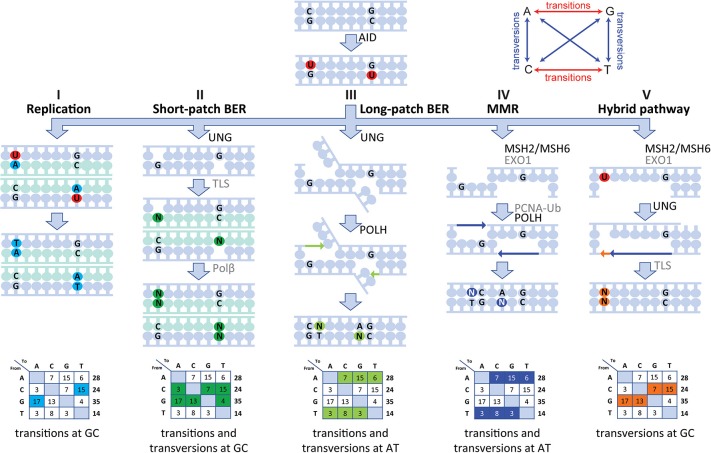
Model of the molecular mechanism of SHM in human. SHM is initiated by AID, which creates a U:G mismatch and can lead to mutations *via five* different routes. **(I)** During replication, the U can be recognized as a T leading to C>T and G>A transversions. **(II)** The U can be recognized and removed by UNG, creating an AP site. During subsequent cell division, this AP site is non-instructive and therefore TLS polymerases incorporate a random nucleotide at this position leading to transition and transversions at GC base pairs. **(III)** A second UNG-dependent pathway involving POLH can be initiated and lead to mutations at AT base pairs. **(IV)** If the U:G mismatch is recognized by the MSH2/MSH6 dimer, multiple bases surrounding the U:G mismatch can be removed by EXO1, creating a single-strand gap. Next, PCNA is polyubiquitinated and recruits POLH to fill this gap, thereby creating mutations at AT base pairs. **(V)** Finally, the MSH2/MSH6-, UNG-hybrid pathway can be initiated with two U's that are present in close proximity to each other but on opposing strands. Here, one of the U's is recognized by MSH2/MSH6 and processed to create a single-strand gap. The other U is processed by UNG, thereby creating an AP site opposite of the single-stranded gap. Next, when the single-stranded gap is filled, a TLS polymerase is recruited to fill the gap opposing the AP site, thereby leading to MSH2/MSH6-dependent transversions at GC base pairs.

Our human data are in line with a five-pathway model to resolve the U lesion introduced by AID (Figure 5) (5, 6, 8, 10, 14, 18). First, if the cell uses the U as a template during replication, the incorporated U is recognized as a T, therefore creating C>T and G>A transitions (Figure 5I). Second, the U can be recognized and removed by UNG, leaving an AP site. During subsequent cell divisions, this AP site is non-instructive, leading to the recruitment of TLS polymerases, including (based on mouse data) REV1 (45), which bypasses the AP site by incorporating a random nucleotide or selectively a C in the case of REV1. This leads to both transitions and transversions at GC base pairs (Figure 5II). Third, our data indicate the presence of a second UNG-dependent pathway in which POLH is involved, which leads to mutations at AT base pairs and therefore is likely based on long-patch BER as the initial lesions lie upstream (Figure 5III). Fourth, if the U:G mismatch is recognized by the MSH2/MSH6 dimer, a stretch of nucleotides surrounding the mismatch is removed by most likely EXO1 (based on mouse models), leaving a single-strand DNA gap. Consecutively, this gap is filled by POLH, which preferentially introduces errors at AT base pairs (Figure 5IV). The fifth and final pathway is dependent on both MSH2/MSH6 and UNG and requires at least two U's on opposing strands in close proximity. On one strand, the U is processed by MSH2/MSH6 and a single-strand gap surrounding the mismatch is created most likely by EXO1 (based on mouse data). If, on the opposing strand, the U is excised by UNG and an AP site is created, TLS polymerases including REV1 (based on mouse data) can bypass the abasic site, while creating transversions and transitions at the AP site (Figure 5V).

The role of PMS2 during SHM is still not completely clear. Recently, Girelli Zubani et al. showed that Ung/Pms2 double knockout mice have a 50% reduction in the number of mutations at AT base pairs (24). They suggest that the Pms2/Mlh1 complex provides the nick required for AT mutagenesis, and that in the absence of the Pms2/Mlh1 complex, Ung can compensate for its function. In this study, we showed that PMS2 deficiency in humans also leads to a reduction of mutations at AT base pairs, as well as to the number of transversion mutations at GC base pairs (Figure 2). Since MSH2 and MSH6 are involved in the hybrid pathway, which results in transversion mutations at GC base pairs, it is tempting to speculate that PMS2 also provides the nick in this fifth pathway (Figure 5). Unfortunately, we were not able to analyze cells from patients with MLH1 deficiency, but it would be very interesting to see if they display the same changes in SHM patterns as the PMS2-deficient patients.

The PMS2-deficient patients had a decreased A/T ratio of 1.5 (range 1.4–1.5), which is in contrast to earlier findings in *Pms2* knockout mice in which an A/T ratio of 4 was reported (24, 43). This would suggest that in the PMS2-deficient patient, error-prone repair is skewed toward the transcribed strand (2:1), while the A/T ratio in mice suggests that the error-prone repair is solely found on the non-transcribed strand. Based on the mouse data, Girelli Zubani et al. have proposed that Pms2/Mlh1 has no strand bias in nick creation and that the strand bias found in wt mouse is the result of other glycosylases only targeting the non-transcribed strand. The reduction in A/T ratio in PMS2 patients found in this study does not comply with the sole targeting of the other glycosylases toward the non-transcribed stand. In contrast, our data show that in the absence of PMS2, AT mutations are more skewed toward the transcribed strand (from 3:1 to 2:1). In addition, the reduction of the A/T ratio is also in contrast with the proposed equal targeting of both strands by PMS2. This discrepancy can be due to the fact that the PMS2-deficient patients might have some residual PMS2 activity. However, four out of the five PMS2 mutations lead to premature stop codons (Table 1), and even though the patients have different mutations, the results were exactly the same for all patients. Therefore, it is likely that the role of PMS2 in mice and humans is slightly different during SHM.

In conclusion, this study is the first to apply NGS on a cohort of genetically well-defined patients suffering from rare genetic defects in *UNG* and MMR. The results obtained in this unique cohort are most compatible with the five-pathway model for the mechanism of SHM based on human data. In addition, our data indicate differences in the role of MSH2 and PMS2 in strand targeting of SHM between mice and man. Our findings emphasize the importance of confirmation of findings in mice in a human setting. Moreover, the here developed method combining NGS and ARGalaxy analysis of BCR mutation data forms the basis for efficient SHM analyses of other related deficiencies.

## Data Availability

The raw data supporting the conclusions of this manuscript will be made available by the authors, without undue reservation, to any qualified researcher.

## Author Contributions

HI, PvS, MvdB, and HJ designed the research and wrote the paper. HI and IP-K performed the experiments. HI and PvS analyzed the data. JL, LB, GD, CB, MS, DJ-L, AA, and MGS provided patient material and critically read the manuscript.

### Conflict of Interest Statement

The authors declare that the research was conducted in the absence of any commercial or financial relationships that could be construed as a potential conflict of interest.
