# Supplementary material for: Repertoire Sequencing of B Cells Elucidates the Role of UNG and Mismatch Repair Proteins in Somatic Hypermutation in Humans
Source: Front Immunol. 2019 Aug 27;10:1913. doi: 10.3389/fimmu.2019.01913 (PMC6718458; doi:10.3389/fimmu.2019.01913)
Supplement: Supplementary file 1 [file Table_1.pdf]

Supplemental Table 1. Overview of mutations and nucleotides sequenced

	AGE	Genetic defect	number of sequences	mutations	number of sequenced A	Number of sequenced C	Number of sequenced T	Number of sequenced G	number of IGHA sequences*	number of IGHG sequences*	A>C	A>G	A>T	C>A	C>G	C>T	G>A	G>C	G>T	T>A	T>C	T>G	RGYW	WRCY	WA	TW
MSH2-01	4	MSH2	246	1565	13000	13483	11022	14835	30	216	22	68	39	77	103	427	453	215	70	24	54	12	558	322	56	48
MSH6-01	16	MSH6	726	8978	39105	38487	33099	43837	270	455	148	459	226	344	635	###	###	###	402	171	284	114	2807	1817	409	361
MSH6-02	10	MSH6	285	2369	15485	14918	13066	17172	85	198	37	119	48	74	165	575	729	360	121	42	69	30	841	488	107	97
MSH6-03	7	MSH6	317	2163	16958	17353	14053	19113	139	178	41	80	44	73	136	585	648	321	103	49	66	17	766	444	85	74
PMS2-01 IGH	14	PMS2	1388	1447	75017	74322	62817	83548			52	189	62	42	94	272	333	160	60	43	110	30	422	283	160	107
PMS2-02	7	PMS2	568	3861	30627	30472	25877	34135	305	263	155	482	175	119	259	747	815	481	170	112	267	79	1082	704	432	294
PMS2-03	10	PMS2	254	1426	13743	13391	11604	15374	132	121	45	155	62	46	120	282	298	200	57	39	86	36	435	247	152	119
PMS2-04	9	PMS2	496	3635	26482	26781	22424	29810	255	239	131	455	194	113	276	682	751	441	159	118	243	72	1082	670	432	294
PMS2-05	14	PMS2	348	2114	18863	18746	15659	20954	228	119	99	257	110	58	146	354	423	318	98	55	154	42	635	351	257	170
UNG02	22	UNG	77	857	4248	4085	3494	4580	51	24	44	69	24	9	9	281	307	18	15	18	45	18	202	169	85	53
NWK33 (3y)	3	Control	291	3274	15545	15808	13068	17511	150	139	215	462	237	83	228	487	590	440	137	77	232	86	828	537	472	288
NWK59 (3Y)	3	Control	554	3236	29634	30101	25008	33269	300	254	212	453	189	105	233	474	519	425	140	130	265	90	706	512	464	305
NWK31 combined (4Y)	4	Control	101	1156	5450	5412	4551	6081	65	36	88	159	63	25	87	159	190	163	64	40	88	30	294	181	166	101
NWK54 (4Y)	4	Control	220	2614	11814	11618	10065	13307	128	90	160	371	155	65	142	403	531	358	110	62	186	71	649	382	363	220
NWK65 combined (4Y)	4	Control	149	1743	7896	8043	6741	8949	88	60	114	251	136	44	106	220	286	252	64	56	166	48	425	258	262	172
NWK56 (6y)	6	Control	300	4182	16379	15904	13610	18082	200	101	287	582	319	119	273	568	758	610	169	128	269	100	1059	652	589	366
NWK64 combined (7.8y)	7.8	Control	98	1274	5274	5262	4465	5893	69	28	77	186	91	42	86	183	207	163	45	49	120	25	328	223	182	132
NWK61_combined (8,8y)	8.8	Control	61	878	3632	3569	3027	4069	55	12	38	149	46	18	52	136	166	113	46	40	60	14	210	142	120	71
NWK66 combined(9,9y)	9.9	Control	126	2114	6700	6873	5710	7525	76	47	143	318	130	54	143	329	334	304	78	55	163	63	518	322	291	183
NWK57 (14y)	14	Control	376	5592	20057	20553	17030	22481	177	197	391	797	350	184	375	845	939	698	235	182	431	164	1303	899	766	507
NWK42 combined (15,6)	15.6	Control	238	3849	12868	12667	10836	14305	106	130	259	546	242	109	266	516	656	506	155	119	338	137	929	589	576	369
NWK5 combined (15,3)	15.3	Control	138	2514	7254	7667	6183	8223	103	34	196	391	158	71	158	386	413	324	96	69	180	72	594	415	364	205
NWK53 (18Y)	18	Control	838	12141	45550	44619	38016	50372	405	423	864	###	809	340	677	###	###	###	498	388	991	380	2897	1781	###	###
NWK43 (20)	20	Control	452	8131	24269	24159	20549	27193	221	224	547	###	557	238	558	###	###	###	355	281	631	253	1819	1262	###	756
NWK303 (22y)	22	Control	1355	19595	73624	71770	61644	81904	812	545	###	###	###	593	###	###	###	###	857	553	###	497	4505	2986	###	###

* patients or controls in which the number of sequences was <45 were excluded from the analysis of the percentage of SHM and subclass distribution. These numbers are indicated in red.
