# Supplementary material for: Repertoire Sequencing of B Cells Elucidates the Role of UNG and Mismatch Repair Proteins in Somatic Hypermutation in Humans
Source: Front Immunol. 2019 Aug 27;10:1913. doi: 10.3389/fimmu.2019.01913 (PMC6718458; doi:10.3389/fimmu.2019.01913)
Supplement: Supplementary file 3 [file Data_Sheet_1.pdf]

Supplemental methods

Title: Repertoire sequencing of B cells elucidates the role of UNG and mismatch repair proteins in somatic hypermutation in humans

Authors: Hanna IJspeert, PhD[1,2*] Pauline A. van Schouwenburg, PhD[1*] Ingrid Pico-Knijnenburg, BSc[2] Jan Loeffen, MD, PhD[3] Laurence Brugieres, MD, PhD[4] Gertjan J. Driessen, MD, PhD[5] Claudia Blattmann, MD, PhD[6] Manon Suerink, MD, PhD[7] Danuta Januszkiewicz-Lewandowska, MD, PhD[8] Amedeo A. Azizi, MD, PhD[9] Marcus G. Seidel, MD, PhD[10] Heinz Jacobs, PhD[11] Mirjam van der Burg, PhD[2]

[1]Department of Immunology, Erasmus MC, University Medical Center Rotterdam, Rotterdam, The Netherlands
[2]Department of Pediatrics, Laboratory for Immunology, Leiden University Medical Center, Leiden, The Netherlands
[3]Department of Pediatric Oncology and Hematology, Sophia Children's Hospital, Erasmus Medical Centre, Rotterdam, the Netherlands
[4]Department of Pediatric and Adolescent Oncology, Gustave Roussy Cancer Campus, Villejuif, France
[5]Department of Paediatrics, Juliana Children's Hospital/Haga Teaching Hospital, The Hague, The Netherlands
[6]Department of Pediatric Hematology and Oncology, Palliative Care, Olgahospital Klinikum Stuttgart, Stuttgart, Germany
[7]Department of Clinical Genetics, Leiden University Medical Center, Leiden, Netherlands
[8]Department of Pediatric Oncology, Hematology and Transplantology, Poznan University of Medical Sciences, Poznan, Poland
[9]Department of Pediatrics and Adolescent Medicine, Medical University Vienna, Vienna, Austria
[10]Research Unit Pediatric Hematology and Immunology, Division of Pediatric Hematology-Oncology, Department of Pediatrics and Adolescent Medicine, Medical University Graz, Graz, Austria
[11]Division of Tumor Biology and Immunology, The Netherlands Cancer Institute, Amsterdam, The Netherlands

Corresponding author:
Mirjam van der Burg, PhD
e-mail: M.van_der_Burg@lumc.nl

Supplemental Methods

Calculations

Figure 1A. Median % of SHM: for every rearrangement the number of mutations in the CDR1-FR3 region were divided by the number of nucleotides in the CDR1-FR3 region. The median percentage for every control or patient is displayed.

Figure 1B. Frequency of IGH subclasses: The frequency of IGHG subclasses was calculated by dividing the number of IGHG1, IGHG2, IGHG3, or IGHG4 by the total number of IGHG rearrangements. The frequency of IGHA subclasses were calculated by dividing the number of IGHA1 or IGHA2 rearrangements by the total number of IGHA rearrangements.

Figure 2A. Frequency of mutations: for all the controls and patients the frequency of a certain mutation (e.g. A>T) was calculated by taken the total mutations of that type divided by the total number of mutations. The frequencies displayed in the transition tables are the averages for every group (HC n=15, UNG n=1, MSH2 n=1, MSH6 n=3, PMS2 n=5) .

Figure 2B. Transversion mutations at GC: The transversion mutation at G were calculated by taking the total transversion mutations at GC divided by the total number of mutations at G and C positions. The transversion mutation at C were calculated by taking the total transversion mutations at GC divided by the total number of mutations at G and C positions.

Figure 2C. Mutations at AT: The transversion at T, transition at T, transversion at A, transition at A mutations were calculated by taken the number of that type of mutation divided by the total number of mutations.

Figure 2D and E. Mutations in WA/TW and RGYW/WRCY motives: The percentage of mutations in WA, TW, RGYQ or WRCY motives were calculated by taken the number of mutations in that motif divided by the total number of mutations.

Figure 2F. Absolute mutation frequency: For every control and patient the number of transitions at GC positions, transversions at GC positions, and transitions plus transversion at AT positions were divided by the total number of sequenced based (in the CDR1-FR3 region). The bars in Figure 2F display the average percentage for every group. Since the absolute mutations frequency is age dependent (see Figure 1A) we divided the HC's into two groups (HC 3-4y n=5, HC 6-22y n=10, UNG n=1, MSH2 n=1, MSH6 n=3, PMS2 n=5).

Figure 3A. Frequency of mutations: For every control and patient the corrected number of A, T, C and G present in the rearrangements was calculated by taking the number of sequenced nucleotides (e.g. A) plus all the number of mutations at that particular nucleotide minus the number of mutations that lead to that particular nucleotides (e.g. total number of sequenced A – (A>C – A>G – A>T + G>A + T>A). Subsequently the correction factor for each nucleotides was calculated by dividing the corrected number of that nucleotide by the total number of nucleotides. This correction factor was applied to all the specific

mutations. So the correction factor for A's was applied to A>C, A>G, and A>T etc. Finally for every control and patient the frequency of a certain mutation was calculated by dividing the corrected number of mutations by the total number of corrected mutations. The percentages in the transition tables are the average of every group (HC n=15, UNG n=1, MSH2 n=1, MSH6 n=3, PMS2 n=5) .

Figure 3B. A/T ratio corrected: For every control and patient the ratio between the number of mutation at A positions divided by the corrected number of A nucleotides and the number of mutations at T positions divided by the corrected number of T nucleotides was calculated.

Figure 4A Transversions at GC positions: The corrected number of mutations and sequenced nucleotides were taken from literature or calculated as described above. For all samples the number of transition mutations at G and C locations were divided by the total number of mutations at G and C locations.

Figure 4B. Mutations at AT base pairs: The corrected number of mutations and sequenced nucleotides were taken from literature or calculated as described above. For all samples the number of mutations at A and T locations were divided by the total number of mutations.

Figure 4C. A/T ratio: The corrected number of mutations and sequenced nucleotides were taken from literature or calculated as described above. For all samples the frequency of mutations at A locations were divided by the frequency of mutations at T locations.
